# Coffee and energy drink use patterns in college freshmen: associations with adverse health behaviors and risk factors

**DOI:** 10.1186/s12889-022-13012-3

**Published:** 2022-03-26

**Authors:** Dace S. Svikis, Pamela M. Dillon, Steven E. Meredith, Leroy R. Thacker, Kathryn Polak, Alexis C. Edwards, David Pomm, Danielle Dick, Kenneth Kendler, Spit for Science Working Group, Danielle M. Dick, Kimberly Pedersen, Zoe Neale, Nathaniel Thomas, Amy E. Adkins, Nathaniel Thomas, Zoe Neale, Kimberly Pedersen, Thomas Bannard, Seung B. Cho, Amy E. Adkins, Peter Barr, Holly Byers, Erin C. Berenz, Erin Caraway, Seung B. Cho, James S. Clifford, Megan Cooke, Elizabeth Do, Alexis C. Edwards, Neeru Goyal, Laura M. Hack, Lisa J. Halberstadt, Sage Hawn, Sally Kuo, Emily Lasko, Jennifer Lend, Mackenzie Lind, Elizabeth Long, Alexandra Martelli, Jacquelyn L. Meyers, Kerry Mitchell, Ashlee Moore, Arden Moscati, Aashir Nasim, Zoe Neale, Jill Opalesky, Cassie Overstreet, A. Christian Pais, Kimberly Pedersen, Tarah Raldiris, Jessica Salvatore, Jeanne Savage, Rebecca Smith, David Sosnowski, Jinni Su, Nathaniel Thomas, Chloe Walker, Marcie Walsh, Teresa Willoughby, Madison Woodroof, Jia Yan, Cuie Sun, Brandon Wormley, Brien Riley, Fazil Aliev, Roseann Peterson, Bradley T. Webb

**Affiliations:** 1 Institute for Women’s Health, Virginia Commonwealth University, 730 E. Broad Street, Richmond, VA 23298 USA; 2 Department of Psychology, Virginia Commonwealth University, 806 W. Franklin St, P.O. Box 842018, Richmond, VA 23284 USA; 3 Center for Clinical and Translational Research, Virginia Commonwealth University, 1200 East Clay St, Richmond, VA 23298 USA; 4 Behavioral Pharmacology Research Unit. Department of Psychiatry and Behavioral Sciences, Johns Hopkins University School of Medicine, 5510 Nathan Shock Dr, Baltimore, MD 21224 USA; 5 Department of Biostatistics, Virginia Commonwealth University, 1100 East Leigh Street, Richmond, VA 23298 USA; 6 Department of Psychiatry, Virginia Institute for Psychiatric and Behavioral Genetics, Virginia Commonwealth University, 1200 East Broad Street, Richmond, VA 23298 USA; 7 Department of Psychiatry, Robert Wood Johnson Medical School, Rutgers University, New Jersey, USA; 8 Department of Psychiatry, Virginia Commonwealth University, 1200 East Broad Street, Richmond, VA 23298 USA

**Keywords:** Caffeine, Energy drinks, Coffee, Alcohol, College students, Smoking, Family history

## Abstract

**Background:**

Public health concern over college students mixing caffeine-containing energy drinks (EDs) and alcohol has contributed to an array of ED-focused research studies. One review found consistent associations between ED use and heavy/problem drinking as well as other drug use and risky behaviors (Nutr Rev 72:87–97, 2014). The extent to which similar patterns exist for other sources of caffeine is not known. The present study examined associations between coffee and ED consumption and alcohol, tobacco and other drug use; alcohol use problems; and parental substance abuse and mental health problems in a sample of college freshmen.

**Methods:**

Subjects were *N* = 1986 freshmen at an urban university who completed an on-line survey about demographics; caffeine; alcohol, tobacco and other drug use; and family history. The sample was 61% female and 53% White. Chi-square analyses and multivariable binary or ordinal logistic regression were used to compare substance use, problem alcohol behavior, and familial risk measures across 3 caffeine use groups: ED (with or without Coffee) (ED + Co; *N* = 350); Coffee but no ED (Co; *N* = 761); and neither coffee nor ED (NoCE; *N* = 875) use.

**Results:**

After adjusting for gender and race, the 3 caffeine use groups differed on 8 of 9 symptoms for alcohol dependence. In all cases, the ED + Co group was most likely to endorse the symptom, followed by the Co group and finally the NoCE group (all *p* < .002). A similar pattern was found for: use 6+ times of 5 other classes of drugs (all *p* < .05); extent of personal and peer smoking (all *p* < .001); and paternal problems with alcohol, drugs and anxiety/depression as well as maternal alcohol problems and depression/anxiety (*p* < .04).

**Conclusions:**

The response pattern was ubiquitous, with ED + Co most likely, Co intermediate, and NoCE least likely to endorse a broad range of substance use, problem alcohol behaviors, and familial risk factors. The finding that the Co group differed from both the ED + Co and NoCE groups on 8 measures and from the NoCE group on one additional measure underscores the importance of looking at coffee in addition to EDs when considering associations between caffeine and other risky behaviors.

## Background

While caffeinated energy drink (ED) use has been linked to numerous physical and mental health problems, the popularity of ED use worldwide continues to rise [1]. In the US, since 2011, ED sales have shown steady growth, with sales in 2016 exceeding $2.8 billion [2]. ED use is most prevalent in adolescents and young adults, with one-third to one-half of adolescents and college students reporting recent (past month) ED use [3–6]. These are the age groups often targeted by aggressive ED marketing efforts [7].

In the US, early ED research was kindled by the landmark study of O’Brien and colleagues (2008) who examined the consumption of EDs mixed with alcohol, which was gaining popularity on college campuses across the country [8]. The investigators found that college drinkers who mixed alcohol with EDs (AMED) were at greater risk for alcohol-related consequences than non-AMED drinkers, even after adjusting for amount of alcohol consumed. It should be noted, however, that the investigators did not assess or adjust for group differences in other sources of caffeine intake [8].

Subsequent cross-sectional studies focused almost exclusively on EDs as a source of caffeine and showed that ED use alone was associated with alcohol, tobacco, and drug use and other risky behaviors [1]. In a review of the literature, Arria et al. (2014) [9] summarized studies showing correlations between ED consumption and: alcohol use [4, 10–17], symptoms of Alcohol Use Disorder [11, 15], tobacco use [4, 10, 12, 14, 16], illicit drug use [4, 10, 12, 14, 16, 18, 19], nonmedical use of prescription drugs [10, 12, 14, 20], other risky behavior (sexual risk taking, fighting, not wearing a seatbelt, etc.) [14, 21], and poor nutrition habits [18].

Given the cross-sectional nature of this research, the mechanisms governing the associations between ED use and other risky behaviors are unknown. Some researchers have suggested that ED consumption is one of many activities associated with a broader pattern of risk-taking behavior [14]. Advertising campaigns that tout the stimulant effects of EDs and, in some cases, glorify drug use may help promote the use of EDs among risk takers or sensation seekers who are more likely to use drugs [22].

The key to the relationship between ED use and other risky behavior, however, might not be ED consumption, per se. Rather, it might lie in the main psychoactive ingredient in EDs—caffeine. Some research has shown that heavy caffeine use (i.e., coffee, tea, and/or soft drinks) and caffeine dependence are associated with dependence on alcohol and illicit drugs [23]. This is significant because in a recent survey of college students, it was coffee, in fact, that was reported as the most widely consumed caffeinated product with almost three-fourths of students (72%) reporting past-year use [24]. Kelpin et al. (2018), using survey data collected prior to ED popularity in the USA, found college females who were daily coffee drinkers were more likely than non-daily coffee users to report heavy alcohol use and a variety of alcohol-related problems [25].

The present study, then, examined associations between coffee and ED consumption and other substance use and related problems in a sample of college freshmen to better understand the significance of caffeine source on observed associations between caffeine intake and adverse health behaviors. Study variables included alcohol and other drug use and related problems, self and peer cigarette smoking, and parental drug and alcohol abuse and mental health concerns. We hypothesized that students reporting ED consumption with or without Coffee (ED + Co) would be most likely to use and report problem behaviors, followed by those consuming only coffee (Co) and finally those reporting no use of either substance (NoCE).

## Methods

### Participants

Participants were college students attending an urban university and participating in the Spit for Science study (see Dick et al., 2014) [26]. They were drawn from an initial pool of *N* = 2056 college freshmen who completed an on-line survey and provided a saliva DNA sample in the fall of 2011. Seventy subjects were subsequently dropped from analyses because of missing data for gender (*N* = 12); caffeine use (*N* = 55) or both (*N* = 3), yielding a final sample of *N* = 1986.

### Procedure

Students were initially informed via campus email about the Spit for Science study. They were told the 15–30-min survey focused on personality and behavior, as well as family, friends, and experiences growing up. For students interested in the study, the email message also contained a link to an on-line survey, where they were given additional information about the study. Informed consent was obtained from students who chose to participate, using Institutional Review Board approved procedures. Compensation ($10 and a “Spit for Science” T-shirt) was dispensed at a central location on-campus, at which time students were invited to provide a saliva DNA sample for an additional $10 (for a more detailed description of Spit for Science study procedures, see Dick et al., 2014) [26].

### Measures

The on-line survey was designed to collect broad-based data on substance use (including caffeine) and problems as well as mental health symptoms, personality traits and various risk and protective factors. Study data were collected and managed using REDCap electronic data capture tools hosted at Virginia Commonwealth University [27]. When possible, standardized measures were used for data collection. The present study analyzed survey responses from the following domains:

#### Demographics

Variables included age, gender and race/ethnicity (dichotomized into White vs Non-White).

#### Caffeine use

Participants were asked about recent consumption of caffeine (“In the last month, in a typical week, on how many days did you drink …” ). The present study focused specifically on coffee, EDs (e.g., Red Bull, Monster, AMP), and energy shots (e.g., 5-Hour Energy). Coffee drinkers were defined as those reporting coffee consumption 1 or more days per week, and caffeinated ED users were defined as those reporting ED use (EDs and/or shots) 1 or more days per week.

#### Alcohol use

Participants were asked to classify their current alcohol use into one of seven categories, which, for purposes of analyses, were collapsed into four groups: Non-users (abstainers); Minimal Users (infrequent and light drinkers); Moderate Users (moderate drinkers) or Heavy/Problem Users (heavy drinkers + problem drinkers + former problem drinkers).

#### Alcohol problems

Symptoms of alcohol dependence were assessed with nine questions adapted from the Semi-Structured Assessment of the Genetics of Alcoholism [28]. Two items had yes/no response options: a) Strong desire to drink or drank too much in situations where alcohol was not permitted; and b) Tolerance (need to drink more alcohol to get the same effect). The other 7 items had three response options (never; 1–2 times; > 3 times) and focused on: wanting to stop drinking; drinking despite self-promise not to drink or drinking more than intended**;** getting drunk when did not want to; stopping or cutting back on important activities to drink; spending several days drinking or recovering from the effects; continuing to drink despite knowing it was causing physical or mental problems; and having withdrawal symptoms (feeling sick for several days after stopping regular drinking). Responses for these 7 items were subsequently dichotomized into No (the symptom never happened) or Yes (it happened 1 or more times).

#### Other drug use

Participants were asked whether they had used each of the following drugs or classes of drugs (illicit or non-medical) 6 or more times in their lives: cannabis, sedatives, stimulants, cocaine, and opioids. Non-medical use was defined as “use without a doctor’s prescription, in greater amounts than prescribed, or for other reasons than those recommended by a doctor.”

#### Tobacco use

Respondents were asked how many cigarettes they had smoked in their lifetime. Responses were classified into 3 groups: 0 cigarettes (never smoked); 1–99 cigarettes; or > 100 cigarettes. Peer smoking was assessed using items from the Monitoring the Future survey [29]. Specifically, participants were asked to think about friends they saw regularly and spent time with (in or outside of school) during the past year and describe the extent to which such friends smoked. Initial response items were combined to create 3 categories: None of them; A Few or Some of them; and Most or All of them.

#### Parental history

Participants were asked whether they thought their biological mother and father had ever experienced problems (yes/no) separately for alcohol, other drugs, and depression/anxiety [26].

#### Coffee/ED use groups

For the present study, self-reported coffee and ED consumption were used to classify *N* = 1986 participants into one of three groups: a) No coffee or ED use (NoCE; *N* = 827); b) Coffee but no ED use (Co; *N* = 761); and c) Use of ED with or without coffee (ED ± Co; *N* = 350). The latter group (ED ± Co) was similar to published literature looking specifically at ED use and included *N* = 266 individuals with ED and coffee use and *N* = 84 individuals with ED use only.

## Results

### Data analysis

Categorical data are presented as percentages while continuous data are presented as mean + SD. Group comparisons for categorical data were performed using the χ^2^ test with the corresponding degrees of freedom, while group comparisons for continuous variables were performed with either a one-way analysis of variance or a non-parametric Kruskal-Wallis test. For all analyses, a *p*-value < 0.05 was considered to be statistically significant. Percentages adjusted for gender and race as well as adjusted odds ratios (OR’s) and 95% confidence intervals for the adjusted OR’s were calculated using either a multivariable binary logistic regression model or an ordinal logistic regression model for variables with more than two levels.

### Demographics

The sample of *N* = 1986 freshmen had a mean age of 18.5 (SD = 0.6) years; approximately one-third was male (38.8%) and half were white (52.7%). Demographic characteristics for the total sample and across the 3 coffee/ED use groups are summarized in Table 1. While the 3 groups were similar in age, they differed on gender (χ^2^ = 87.91, d.f. = 2, *p* < 0.0001) and race (χ^2^ = 19.25, d.f. = 2, *p* < 0.0001). Specifically, there were more females in the Co group (74.5%) as compared to both the NoCE (52.2%) and ED ± Co (54.6%) groups. The 3 groups also differed in racial representation, with a higher percentage of White participants in the ED ± Co (63.0%) as compared to the Co (54.9%) and NoCE (46.6%) groups. Subsequent group comparisons were adjusted to take into account differences in gender and race composition.

**Table 1:** Demographic Data by Coffee/ED Use Group

	Total (***n*** = 1986)	No Coffee (NoCE) (***n*** = 875)	Coffee Only (Co) (***n*** = 761)	ED ± Coffee (ED ± Co) (***n*** = 350)
Age	18.5 (0.6)	18.5 (0.6)	18.5 (0.7)	18.5 (0.6)
Gender % Male	38.8% (771/1986)	47.8% (418/875)	25.5% (194/761)	45.4% (159/350)
White	52.7% (1031/1958)	46.6% (402/863)	54.9% (413/752)	63.0% (216/343)

### Alcohol use and problems by coffee/ED use group

The data for gender and race-adjusted group effect test comparisons of the 3 coffee/ED use groups on measures of alcohol, tobacco and other drug use as well as parental history variables are summarized in Table 2. In addition, adjusted odds ratios are shown for all possible 2-group comparisons (Co to NoCE, ED ± Co to NoCE and ED ± Co to Co).

**Table 2:** Personal, Peer and Family Substance Use and Problems by Coffee/ED Use Group

	Percentages adjusted for Gender and Race				Adjusted OR (95% CI)		
	No Coffee or ED use (***n*** = 875) (NoCE)	Coffee only (no ED) (***n*** = 761) (Co)	Coffee and/or ED Use (***n*** = 350) (CoED)	Group Effect Test ***p***-value	Co Vs. NoCE	Co + ED Vs. NoCE	Co + ED Vs. Co
**Alcohol Use and Problems**							
Alcohol Use				< 0.0001	**1.58 (1.30, 1.91)**	**2.43 (1.91, 3.09)**	**1.54 (1.21, 1.96)**
Non-user	52.7%	41.3%	31.4%				
Minimal User	37.1%	43.4%	46.9%				
Moderate User	9.0%	13.3%	18.7%				
Heavy/Problem User	1.3%	2.0%	3.1%				
Alcohol Problems							
Strong desire to Drink	3.3%	4.8%	7.0%	0.0125	1.47 (0.91, 2.37)	**2.21 (1.31, 3.73)**	1.50 (0.91, 32.46)
Tolerance	6.7%	10.8%	17.5%	< 0.0001	**1.68 (1.24, 2.29)**	**2.94 (2.11, 4.12)**	**1.75 (1.27, 2.41)**
Want to stop Drinking	6.6%	9.4%	15.1%	0.0035	**1.46 (1.04, 2.05)**	**2.49 (1.72, 3.62)**	**1.71 (1.19, 2.45)**
Drank more than Intended	7.5%	12.7%	19.7%	< 0.0001	**1.78 (1.31, 2.42)**	**3.02 (2.15, 4.23)**	**1.70 (1.23, 2.33)**
Became drunk Unintentionally	6.9%	8.1%	13.1%	0.0004	1.19 (0.86, 1.66)	**2.04 (1.42, 2.93)**	**1.71 (1.19, 2.45)**
Reduced activities because of Drinking	3.8%	3.8%	7.9%	0.0011	0.99 (0.62, 1.56)	**2.15 (1.35, 3.43)**	**2.19 (1.36, 3.52)**
Spent several days Recovering from Effects	5.3%	5.8%	10.3%	0.0005	1.10 (0.77, 1.58)	**2.05 (1.40, 3.01)**	**1.86 (1.26, 2.74)**
Use caused physical/mental Problems	3.2%	3.7%	6.7%	0.0075	1.17 (0.72, 1.92)	**2.19 (1.31, 3.68)**	**1.87 (1.13, 3.11)**
Withdrawal	1.7%	1.3%	2.7%	0.1245	0.76 (0.38, 1.51)	1.63 (0.83, 3.18)	2.15 (1.01, 4.54)
**Other Drug Use (Lifetime)**							
Cannabis 6+ Times	14.7%	18.1%	29.5%	< 0.0001	**1.29 (1.02, 1.63)**	**2.43 (1.85, 3.19)**	**1.89 (1.43, 2.49)**
Sedatives 6+ Times	0.9%	1.1%	3.2%	0.0003	1.20 (0.58, 2.49)	**3.51 (1.76, 7.00)**	**2.92 (1.51, 5.64)**
Stimulants 6+ Times	1.3%	2.4%	3.8%	0.0003	**1.89 (1.10, 3.22)**	**3.10 (1.78, 5.40)**	1.64 (0.99, 2.72)
Cocaine 6+ Times	0.5%	0.3%	1.2%	0.0452	0.64 (0.18, 2.26)	2.63 (0.94, 7.38)	**4.10 (1.21, 13.90)**
Opioids 6+ Times	0.7%	0.5%	1.4%	0.0485	0.78 (0.34, 1.77)	2.04 (0.98, 4.27)	**2.62 (1.14, 6.04)**
**Tobacco Use (Lifetime)**							
Smoked 1+ Cigarettes	17.6%	29.0%	38.6%	<.0001	**1.91 (1.53, 2.39)**	**2.94 (2.25, 3.84)**	**1.53 (1.18, 2.00)**
Smoking Quantity				< 0.0001	**1.86 (1.50, 2.31)**	**3.21 (2.48, 4.13)**	**1.73 (1.34, 2.22)**
0 Cigarettes	82.8%	72.2%	60.0%				
1–99 Cigarettes	13.9%	21.9%	30.2%				
100+ Cigarettes	3.3%	5.9%	9.8%				
Number of Friends who Smoke				< 0.0001	**1.58 (1.30, 1.93)**	**2.51 (1.95, 3.24)**	**1.59 (1.23, 2.06)**
None of them	49.7%	38.6%	28.4%				
A few/Some of them	44.9%	53.3%	59.4%				
Most/All of them	5.4%	8.1%	12.2%				
**Parental History**							
Biological Mother							
Alcohol Problems	4.6%	6.0%	8.6%	0.0104	1.32 (0.90, 1.95)	**1.95 (1.26, 3.00)**	1.47 (0.97, 2.23)
Drug Problems	5.1%	5.7%	6.8%	0.4957	1.12 (0.73, 1.72)	1.36 (0.82, 2.26)	1.21 (0.73, 2.03)
Depression/Anxiety	34.2%	38.9%	45.0%	0.0049	1.22 (0.98, 1.53)	**1.57 (1.19, 2.07)**	1.28 (0.97, 1.70)
Biological Father							
Alcohol Problems	21.0%	21.2%	29.5%	0.0004	1.01 (0.78, 1.30)	**1.57 (1.17, 2.12)**	**1.55 (1.15, 2.11)**
Drug Problems	14.1%	13.2%	19.7%	0.0193	0.93 (0.69, 1.25)	**1.50 (1.07, 2.10)**	**1.61 (1.14, 2.29)**
Depression/Anxiety	25.0%	26.8%	32.8%	0.0383	1.10 (0.86, 1.41)	**1.47 (1.09, 2.12)**	1.34 (0.98, 1.81)

(Percentages adjusted for gender and race as well as Adjusted Odds Ratios (OR) and 95% Confidence Intervals (CI))

For alcohol, group effects were found for pattern of alcohol use (None, Minimal users, Moderate users and Heavy/Problem users), with greater moderate and heavy use among the ED ± Co group and higher rates of abstinence in the NoCE group. Group effects were also found for 8 of the 9 symptoms of alcohol use disorder (AUD) and in all but one case, the ED ± Co group was most likely to endorse the item, followed by the Co and finally the NoCE group. For 3 of the symptoms, (tolerance; wanting to stop; consuming more than intended), the Co group was 1.46–1.78 times more likely to endorse the symptom than the NoCE group. The ED ± Co group differed from the NoCE group on all 8 symptoms, with Adjusted Odds Ratios (AORs) ranging from 2.04 (95% CI: 1.42–2.93) for desire to cut down/stop drinking to 3.13 (95% CI: 2.02–4.84) for drinking more than they intended. Finally, the ED ± Co group differed from the Co group on 7 of the 8 symptoms, with AORs ranging from 1.70 (95% CI: 1.23–2.33) for drinking more than they intended to 2.19 (95% CI: 1.36–3.52) for reduced other activities due to drinking.

### Other drug use and problems by coffee/ED use group

For other drug use (6+ times lifetime), significant group effects were found for all 5 classes of drugs. The Co and NoCE groups differed significantly for 2 drug classes, with the Co group 1.29 times more likely than NoCE group to report cannabis use (95% CI: 1.02–1.63) and 1.89 times more likely to report stimulant use (95% CI: 1.10–3.22). The ED ± Co group was more likely than the NoCE group to use 3 of the 5 categories of drugs, with AORs ranging from 2.43 for cannabis (95% CI: 1.85–3.19) to 3.10 for stimulants (95% CI: 1.78–5.40) to 3.51 for sedative/hypnotics (95% CI: 1.76–7.0). Finally, the ED ± Co group was more likely than the Co group to report use of all drugs except stimulants, with AORs ranging from 1.89 for cannabis (95% CI: 1.43–2.49) to 4.10 for cocaine (95% OR = 1.21–13.90).

### Tobacco use by coffee/ED use group

For tobacco, significant group effects were found for all three cigarette smoking variables: smoking at least one cigarette (lifetime), number of cigarettes smoked (lifetime) and proportion of friends who smoke. Specifically, ED ± Co group members were 1.53 times more likely to have ever smoked a cigarette than Co group members (CI: 1.18, 2.0) and Co group members were 1.91 times more likely to have ever smoked than NoCE group members (CI: 1.53, 2.39). The largest difference was found between the ED ± Co and NoCE groups, with ED ± Cos nearly 3 times more likely to report ever smoking than NoCEs (AOR: 2.94; CI: 2.25, 3.84). Similar patterns were seen for smoking quantity (lifetime), with AOR’s ranging from 1.73 (ED ± Co vs CO) to 3.21 (ED ± Co vs NoCE), as well as peer smoking (ED ± Cos most likely and NoCEs least likely to report most/all of their friends smoked).

### Parental problems by coffee/ED use group

The 3 coffee/ED use groups differed in prevalence of maternal alcohol problems and depression/anxiety (.005 < *p* < .01) and paternal alcohol and drug problems and mental health (.004 < *p* < .04). For maternal alcohol problems and depression/anxiety, only the ED + Co group differed from the NoCE group with OR’s ranging from 1.57 for mental health to 1.97 for alcohol problems. For fathers, group effects were found for alcohol problems, with ED + Co group members differing from both the Co (AOR: 1.55; CI: 1.15, 2.11) and NoCE (AOR: 1.17; CI: 1.17, 2.12) groups. Similarly for paternal drug problems, the ED ± Co group differed from both the Co (AOR: 1.61; CI: 1.14, 2.29) and NoCE (AOR: 1.50; CI: 1.07, 2.10) groups. For paternal depression/anxiety, only the ED ± Co group differed from the NoCE group (AOR: 1.47; CI: 1.0 2.12). symptoms.

## Discussion

### Principal findings

Spurred by the steady rise in ED use and the targeted marketing of these drinks to young adults, much of the existing research regarding caffeine use by college students has focused exclusively on EDs and their associations with a variety of risky health behaviors [9]. Studies have shown, however, that other sources of caffeine such as coffee and soft drinks are more frequently used by college students than EDs, and these sources of caffeine should be considered when evaluating associations between caffeine and other substance use and problem behaviors [25]. The present study is among the first to look concurrently at coffee and ED use in college students and to evaluate associations between their use and alcohol, tobacco and other drug use; alcohol use problems; and parental substance abuse and mental health problems. Analyses found students who consumed EDs (with or without concurrent coffee use) were most likely to report other substance use, alcohol-related problem behaviors, and peer/family risk factors for substance use followed by students who consumed coffee only, and finally, students who reported using neither EDs nor coffee. The data are particularly noteworthy for the consistent response pattern observed across almost all domains assessed.

In most previous research, the relationship between other sources of caffeine and adverse health behaviors was either not considered^,14,17,30^ or used as a covariate in the data analysis [11]. The focus on EDs as the singular source of caffeine in these studies started with the compelling data from O’Brien and colleagues (2008) who reported an association between the use of EDs mixed with alcohol and both risky drinking and alcohol-associated adverse health behaviors [8]. Subsequent researchers continued to focus on EDs and risky health behaviors, in part because of the intense marketing efforts ED makers directed at college-age students and the relatively higher amounts of caffeine in EDs compared to traditional sources of caffeine (e.g., 40 mg caffeine in a 12-oz can of Coca-Cola vs 80 mg in a 12-oz can of Red Bull). More recently, however, many specialty coffee drinks (150 mg in a 12-oz cappuccino) and even soft drinks (110 mg caffeine in a 12-oz can of Coke Energy) contain caffeine in amounts like those found in EDs. Another reason researchers focused singularly on EDs was because these beverages often are consumed more rapidly than hot caffeinated beverages like coffee. Many thought the relatively rapid rate of consumption of EDs may lead to higher caffeine levels and thus, greater association with risky health behaviors, compared to caffeinated drinks that are typically consumed more slowly, like hot coffee drinks. White et al. (2016) however, recently showed there was no clinically significant difference in caffeine exposure (i.e., T_max_, MRT, MAT or AUC_0–∞_) regardless of the rapidity with which caffeine was consumed [30].

Much of the early research in college students who mixed EDs with alcohol showed that these students consumed alcohol more frequently, in higher amounts, and with more episodes of binge and problem drinking than students consuming alcohol without ED mixers [8, 30, 31]. Not surprisingly, AmED users also were more likely than non-AmED users to engage in other risky health behaviors including risky sexual behavior, dangerous driving behavior, and physical altercations [8, 32, 33]. Both clinical and laboratory research suggest students who consume AmED have altered perceptions of their levels of intoxication, with these students not recognizing their levels of impairment [8, 34]. Early research also consistently found college students who used ED, independent of concomitant alcohol use, were more likely to report alcohol use; meet criteria for alcohol dependence; use tobacco, marijuana, and nonmedical prescription drugs; and engage in risky sexual and physical behaviors [10, 14, 21].

A few significant exceptions to the early ED-only and AmED-only focused research in college students showed that other sources of caffeine also were associated with risky health behaviors. Thombs and colleagues (2011) compared the effects of AmEDs to alcohol mixed with cola and alcohol alone on alcohol use in college students [35]. The researchers found a dose-dependent relationship between the estimated amount of caffeine consumed from both EDs and soft drinks and risky alcohol use. Using data from a group of Icelandic college students, Kristjansson et al. (2015) showed that daily consumption of coffee, soft drinks, and EDs, but not tea, was positively associated with drinking AmEDs [36]. In addition, Anderson and Juliano (2012) showed that estimated mean weekly caffeine consumption, regardless of the source, was positively correlated with the amount of alcohol consumed by college students [37]. These cross-sectional studies suggest the amount of caffeine consumed is more important than the source of caffeine with regard to the likelihood that college students will engage in adverse health behaviors. More recently, Dillon and colleagues (2019) investigated the relationship between all sources of caffeine and adverse health behaviors in college freshmen [38]. They found that students who consumed caffeine daily from any source were more likely to report alcohol, cigarette, and nonmedical drug use and problem drinking than those who did not consume caffeine.

In the present study, we elected to focus on coffee and ED consumption in college students for three reasons. First, these beverages typically have the highest caffeine content and, over time, they have come to represent a greater proportion of caffeine intake in US children and adolescents [39]. Second, coffee is used frequently by college students [24, 40], with one recent convenience sample survey of college students finding coffee to be their primary source of caffeine intake (72%), followed by soft drinks (69%), tea (61%), and EDs (36%) [24]. Third, research done by our group prior to the surge in popularity of EDs underscored the importance of considering coffee when evaluating the effects of caffeine on substance use. Our research showed that college women who drank coffee daily were more likely to report heavier drinking and alcohol-related problems than non-daily coffee drinkers [25].

Like previous work, this research found an association between caffeine and risky health behaviors. This relationship was more robust for students in the ED + Co group compared with those who drank coffee only and those consuming neither beverage. While the cross-sectional nature of the work limits our ability to establish a causal relationship between caffeine, other substance use, and alcohol use problems, the associations are likely a result of a combination of genetic, psychobiological, and environmental factors.

Our study is among the first to look at familial factors associated with caffeine use. We found participants reporting ED ± Co use were more likely to report maternal alcohol problems and depression/anxiety symptoms as well as paternal alcohol and drug problems and depression/anxiety. Such familial clustering may occur because of both a shared environment and genetic factors. In fact, Kendler, Myers, and Gardner (2006) [23], in a study in adult twins, found that a link between caffeine use and the development of substance use and psychiatric disorders was due primarily to familial factors, including genetic factors. With the compelling and consistent association between EDs and risk-taking behaviors, most frequently other substance use, researchers have linked sensation-seeking personality traits and ED use. College students who scored higher on measures of sensation-seeking were more likely to consume ED and AmED [10, 14, 41]. This may be due to caffeine’s potentiation of the psychostimulant effect of other drugs of abuse through its effects on the adenosine and dopamine pathways. In addition, when combined with alcohol, caffeine blunts the depressant effects and enhances the stimulant effects of alcohol, which alone is associated with risk-taking, by affecting the same pathways [42]. The increased stimulant effect, decreased depressant effects, and propensity for risk-taking may lead to increased sensation-seeking behavior, including ED use.

Environmental factors likely impact the association between caffeine use and risky health behaviors as well. Almost all college students use caffeine regularly [43]. At the same time, most college students are in the age range, late teens and early 20s, at highest risk for the onset of many substance use disorders [26]. The temporal intersection between high frequency caffeine use and increased prevalence of substance use may explain the association between caffeine and risky health behaviors. Patterns between ED use and other drug use have also been found in younger age groups (8th, 10th, and 12th graders) [3]. In addition, alcohol and other substances like marijuana and tobacco are often part of the college milieu, and students may use caffeine to affect the pharmacodynamic effects of these other substances. For instance, students may concurrently consume caffeine to offset the depressant effects of alcohol or marijuana while socializing or use caffeine to increase their energy when they have school obligations after a night of heavy drinking. Finally, there is evidence that peer influence increases adolescents’ substance use, and this may contribute to the risky behaviors reported by our sample [44]. Indeed, we found that students who used EDs and/or coffee (ED ± Co and Co groups) were more likely to report smoking and having friends who smoked than the NoCE group.

### Limitations

There are several limitations to the present study. First, we relied on retrospective self-report data to address our research question. Second, participants were surveyed about recent (past 30 days) caffeine consumption, which did not allow us to examine use patterns over longer periods. Nonetheless, a 30-day timeframe focused on recent caffeine use appeared to be an appropriate starting point for examining substance use/problems associated with cross-beverage caffeine consumption. Third, the low number of ED only (no coffee) users (*N* = 84) prevented statistical power for a 4-group comparison. Instead, present study analyses included an ED + Co group in which three-fourths of the sample reported use of both ED and coffee (76%) and one-fourth reported ED use but no coffee. One advantage of the ED + Co group is that it is similar to much of the published research in which ED use was defined without attention to concurrent coffee use, and this allows our data to be compared to the extant literature. Fourth, only frequency of caffeine use was assessed, with no quantity of use data. The survey used for this research was originally designed to assess alcohol use in college students, with limited caffeine use questions, and future research should collect more detailed quantitative data about quantity and frequency of caffeine use. Fifth, caffeine use was restricted to only coffee and ED use; other sources of caffeine intake (e.g., tea, sodas) were not included.

## Conclusions

The current study presents benchmark data on the elevated risks associated with ED + Co and Co use compared to use of neither substance (NoCE). Specifically, we found a consistent response pattern in which NoCE users were least likely to report substance use and related problem behaviors and ED ± Co users were most likely to endorse such behaviors, with Co users falling in the middle. Whereas the relationship between caffeine use and risky behavior has been previously established [14], the mechanisms underlying these associations are unknown and are likely a confluence of factors. Additional research is needed to disentangle the effects of amount and type of caffeine use from genetic factors, personality traits, and environmental influences that may mediate these adverse health behaviors.

Present study findings have significant public health implications. Caffeine use is ubiquitous on college campuses, and it is associated with a host of substance-related and other risky adverse health behaviors. The present study found relationships between coffee and ED use and other substance use, alcohol-related problems, and several risk factors for alcohol and drug use. These relationships were strongest for the ED group, but coffee consumption was also associated with risky health behaviors. While evaluating regular caffeine use from a variety of sources including coffee and EDs is important for research purposes, the findings from this research unequivocally show that ED use is most significant for identifying students at risk for other substance use and associated adverse health behaviors. With the social acceptability of EDs, screening for regular ED use in college students may provide a non-stigmatizing way to identify students at higher risk for alcohol/drug misuse, and to prioritize them to receive substance use education and intervention.

## Data Availability

Data from this study are available to qualified researchers via dbGaP (phs001754.v2.p1).

## Abbreviations

ED: Energy Drink
ED ± Co: Coffee and Energy Drink Use
Co: Coffee but no Energy Drink Use
NoCE: Neither Coffee nor Energy Drink Use
AmED: Alcohol Mixed with Energy Drinks
AUD: Alcohol Use Disorder
AOR: Adjusted Odds Ratio
OR: Odds Ratio
CI: Confidence Interval
